# Functional equation modeling of adaptive operant-control systems via Matkowski fixed point theory

**DOI:** 10.1371/journal.pone.0339678

**Published:** 2026-01-05

**Authors:** S. Monica, D. Ramesh Kumar

**Affiliations:** Department of Mathematics, School of Advanced Sciences, Vellore Institute of Technology, Vellore, Tamil Nadu, India; University of Anbar, IRAQ

## Abstract

This paper presents a generalized form of the functional equation used in operant-control models by removing the requirement for initial conditions. The proposed formulation extends earlier studies in mathematical psychology and provides a broader analytical framework for modeling operant-control behavior. Using the Matkowski fixed point theorem, we prove the existence and uniqueness of a probabilistic solution to the generalized equation. Illustrative examples and simulations are included to demonstrate the validity of the theoretical results. This work shows that fixed point theory can effectively support the formulation and analysis of control-based behavioral models.

## Introduction

Operant conditioning was first described by psychologist B.F. Skinner [1]. His theory rests on two fundamental assumptions: first, human and animal behavior is influenced by environmental factors; second, the consequences of a behavior determine its likelihood of being repeated. In fact, Behaviors that result in positive consequences are more likely to be repeated, while those that result in negative consequences are less likely to occur again [2–5].

Here, we present two experiments that inspired our investigation in this study. During World War II, B.F. Skinner [6] conducted research on Project Pigeon, also known as Project Orcon, an experimental initiative aimed at developing pigeon guided missiles. Skinner trained pigeons to peck at a target, rewarding them with food upon successful completion of the task. On the other hand, Gregory S. Berns et.al [7] conducted experiments involving water and juice, revealing a notable divergence in brain responses between prediction and subjective preferences [8]. Brain responses to preferences were predominantly cortical, whereas responses to predictability involved activation of reward systems targeted by dopaminergic neurons [9] and [10]. Further, several unresolved issues in this field merit further exploration.

In the separate line of research, the dynamic relationship between predators and their prey is a critical focus in ecosystem studies [11]. Recent research indicates that predation can significantly influence prey population size, acting as a top-down control mechanism. Indeed, observing the interplay between these population dynamics offers insights into long-term changes in population sizes over time [12].

In 1973, Lyubich and Shapiro [13] studied the existence and uniqueness of a continuous solution f:[0,1]→[0,1] of the following functional equation:

$$f(x)=xf((1-a_{1})x+a_{1})+(1-x)f((1-a_{2})x)$$ (1)

for all x∈[0,1], where $0<a_{1}\leq a_{2}<1$ and the functional equation Eq (1) appears in mathematical biology and the theory of learning to observe the nature of predator animals that hunt two kinds of prey. Such a conduct is defined by the Markov process in the state space [0,1] with the probabilities of transition operators *g*_1_ and *g*_2_ are given by

$g_{1}(x)=(1-a_{1})x+a_{1},\;g_{2}(x)=(1-a_{2})x$.

In the mathematical model Eq (1), the solution *f* is the final probability of the event when the predator is fixed on one category of prey, knowing that the initial probability for this category to be chosen is equal to *x* [14]. Also, Turab and Sintunavarat [15] observe the behaviour of the paradise fish in a two-choice situation [16].

In [12], Lyubich and Shapiro used Schauder’s fixed point theorem to prove the existence of a solution of the functional equation Eq (1) of the following form:

$$f(x)=\sum_{i=1}^{\infty }k_{i}x^{i}=0,\;k_{i}\geq 0$$ (2)

satisfying the conditions f(0)=0,f(1)=1.

After this, Istra’tescu [17] proposed the existence and uniqueness result for the solution of the functional equation Eq (1) with condition Eq (2) using the Banach contraction mapping principle.

In this context, Dmitriev and Shapiro [18] found a solution of Eq (1) by a direct method. They denoted $\lambda_{1}=1-a_{1}$ and $\lambda_{2}=1-a_{2}$ and used the substitution

$$f(x)=x+(\lambda_{2}-\lambda_{1})x(1-x)h(x)$$ (3)

to reduce the functional equation Eq (3) with the unknown function *f* to the following functional equation:

$$h(x)=\lambda_{1}(1-\lambda_{1}(1-x))h((1-\lambda_{1}(1-x))+\lambda_{2}(1-\lambda_{2}x)h(\lambda_{2}x)+1$$ (4)

where *h* is an unknown mapping. Thus, they proved that the solution of the functional equation Eq (4) can be presented as

$$h(x)=\sum_{i=1}^{\infty }f_{i}(x)$$ (5)

where


$$f_{0}(x)=\sum_{j=1}^{\infty }\lambda_{2}^{j}\mu_{j}(x,\lambda_{2}),$$

$$f_{i+1}(x)=\lambda_{1}\sum_{j=1}^{\infty }\lambda_{2}^{j}\mu_{j}(x,\lambda_{2})(1-(1-\lambda_{2}^{j}x))f_{i}(1-(1-\lambda_{2}^{j}x))$$

and


$$\mu_{0}(x,\lambda_{2})=1,\;\mu_{j}(x,\lambda_{2})=(1-\lambda_{2}x)......(1-\lambda_{2}^{j}x),\;j\geq 1.$$

Recently, the result of Istra’tescu was expanded by Berinde and Khan [19], who discussed the existence and uniqueness of a solution of the proposed functional equation using the Banach fixed point theorem. They modeled the functional equation Eq (1) in the following form:

$$f(x)=xf(g_{1}(x))+(1-x)f(g_{2}(x))$$ (6)

where f:[0,1]→[0,1] is an unknown mapping, $g_{1}(x),g_{2}(x):[0,1]\to [0,1]$ are contraction mappings satisfying

$$g_{1}(1)=1,g_{2}(0)=0.$$ (7)

In recent times, Turab et al. [15,20–25] introduced new conditions and proved the existence and uniqueness of a solution of the functional equation Eq (6) using one boundary condition in Eq (7).

This paper addresses an important open problem in [25] by applying the Matkowski fixed point theorem under minimal assumptions to a new modified functional equation Eq (8). Equation Eq (8) generalizes Eq (6) to an integral form; in particular, by choosing α(x,t)=x, β(x,t)=1−x, ${ℰ}_{1}(t)=g_{1}(x)$, ${ℰ}_{2}(t)=g_{2}(x)$, and evaluating at *t* = *x*, equation Eq (8) reduces to Eq (6). This shows that the earlier equation is a special case of our generalization, which we then proceed to solve.

Furthermore, we provide a solution to the open problem posed in [25] by proving that, unlike the previous setting, the initial conditions ${ℰ}_{1}(0)=0$ and ${ℰ}_{2}(0)=0$ are not essential for ensuring the existence and uniqueness of solutions. In addition, we present two experiments based on operant-control theory together with illustrative examples.

A key contribution of this work is the generalization of previous functional equations in operant-control models by formulating an integral version and applying the Matkowski fixed point theorem. This approach establishes existence and uniqueness without requiring initial conditions, demonstrates that earlier results are special cases, and provides a broader framework for modeling operant-control behavior.

## Preliminaries

Following definitions and well-known fixed point results will be required in the continuation.

**Definition 1.** *Let X and Y be two nonempty sets and T:X→Y be a single valued mapping. A point x∈X is called a fixed point of T if and only if x = Tx.*

**Definition 2.** *Let (X,d) be a metric space. A mapping T:X→X is called a Banach contraction mapping if there is a non-negative real number α<1 such that for all x,y∈X,*


d(Tx,Ty)≤αd(x,y).

**Theorem 1** ( **Matkowski fixed point theorem**). *Let (X,d) be a complete metric space and suppose T:X→X satisfies for all x,y∈X,*


d(Tx,Ty)≤φ(d(x,y))

*where φ:[0,∞)→[0,∞) is non-decreasing and right-continuous such that φ(t)<t for all t>0. Then T has a unique fixed point $x^{*}\in X$, i.e., there exists a unique $x^{*}\in X$ such that $Tx^{*}=x^{*}$.*

*Moreover, for any x∈X, the sequence {T* ^*n*^ *x} converges to the fixed point $x^{*}$.*

Fixed point theory is instrumental in solving functional equations due to its ability to transform complex problems into simpler ones by identifying points where a mapping maps to itself. This theory is key in several applications, such as iterative methods like the Picard iteration, which use fixed points to find solutions to differential and integral equations. Fixed point theorems often serve as existence proofs for solutions to various functional equations, and many problems in nonlinear analysis and differential equations rely on fixed point results to establish the existence of solutions.

## Main results

Let 𝒳 be a collection of all continuous real-valued mappings ℰ:[0,1]→ℝ such that ℰ(0)=0.

If ‖.‖:𝒳→[0,∞) is defined by $\left\|ℰ\|=\underset{0\leq x\leq 1}{\sup}|ℰ(x)\right|$ for all ℰ∈𝒳, then (𝒳,‖.‖) is a Banach space. Indeed, completeness follows because 𝒳 is a closed subspace of the classical Banach space $\left(C([0,1]),\|\cdot {\|}_{\infty }\right)$, since the condition ℰ(0)=0 defines a closed subset.

Furthermore, we shall be interested in the existence and uniqueness of a solution to the following functional equation:

$$\Gamma (x)=\int_{0}^{x}\alpha (x,t)\Gamma ({ℰ}_{1}(t))+\beta (x,t)\Gamma ({ℰ}_{2}(t))dt,$$ (8)

where α(x,t) and β(x,t) are non-negative and continuous mappings such that


α(x,t)+β(x,t)=1,0≤t≤x≤1,

${ℰ}_{1}$ and ${ℰ}_{2}$ are continuous mappings from [0,1] to [0,1], and Γ:[0,1]→ℝ is the unknown mapping with the initial condition Γ(0)=0.

We now turn to our main results in this paper.

**Theorem 2.** *Consider the functional equation* Eq (8)*. Suppose that ${ℰ}_{1},{ℰ}_{2}$ are Banach Contraction mappings with contraction coefficients $\kappa_{1},\kappa_{2}$ respectively and satisfy $\kappa_{1}+\kappa_{2}<1.$ Then* Eq (8) *has a unique solution. Moreover, the sequence $\left\{\Gamma_{n}\right\}$ in 𝒳 defined for each x∈[0,1] by*

$$\Gamma_{n}(x)=\int_{0}^{x}\alpha (x,t)\Gamma_{n}({ℰ}_{1}(t))+\beta (x,t)\Gamma_{n}({ℰ}_{2}(t))dt,$$ (9)

*for all n∈ℕ, where $\Gamma_{0}$ is given in 𝒳, converges to a unique solution of* Eq (8).

**proof.** Define an operator T:𝒳→𝒳 by


$$(T\Gamma )(x)=\int_{0}^{x}\alpha (x,t)\Gamma ({ℰ}_{1}(t))+\beta (x,t)\Gamma ({ℰ}_{2}(t))dt.$$

Here, the mapping *T* maps any continuous mapping Γ to a new mapping TΓ defined by the integral above.

Since ${ℰ}_{1}$ and ${ℰ}_{2}$ are continuous mappings from [0,1] to [0,1], and Γ∈𝒳 is continuous on [0,1], the compositions


$$\Gamma ({ℰ}_{1}(t))\;\text{and}\;\Gamma ({ℰ}_{2}(t))$$

are also continuous on [0,1]. Hence, the integrand


$$\alpha (x,t)\Gamma ({ℰ}_{1}(t))+\beta (x,t)\Gamma ({ℰ}_{2}(t))$$

is continuous in both variables *x* and *t* on the region 0≤t≤x≤1. Therefore, the integral expression defining (TΓ)(x) is continuous with respect to *x*, and in particular,


(TΓ)(0)=0.

Thus, TΓ∈𝒳, and so *T* is a well-defined operator on 𝒳.

Next, we show that *T* satisfies the conditions of the Matkowski fixed point theorem. Define a mapping φ:[0,∞)→[0,∞) by


$$\varphi (t)=(\kappa_{1}+\kappa_{2})t,$$

where $\kappa_{1}$ and $\kappa_{2}$ are the contraction constants of the mappings ${ℰ}_{1}$ and ${ℰ}_{2}$ respectively.

Clearly, the mapping $\varphi (t)=(\kappa_{1}+\kappa_{2})t$ is non-decreasing and right-continuous.

Consider two mappings $\Gamma_{1},\Gamma_{2}\in 𝒳$. For any x∈[0,1], we have


$$|(T\Gamma_{1})(x)-(T\Gamma_{2})(x)|=|\int_{0}^{x}\alpha (x,t)[\Gamma_{1}({ℰ}_{1}(t))-\Gamma_{2}({ℰ}_{1}(t))]\;\;+\beta (x,t)[\Gamma_{1}({ℰ}_{2}(t))-\Gamma_{2}({ℰ}_{2}(t))]\;dt|.$$

Using the triangle inequality, this can be bounded by


$$|(T\Gamma_{1})(x)-(T\Gamma_{2})(x)|\leq \int_{0}^{x}|\alpha (x,t)|\cdot |\Gamma_{1}({ℰ}_{1}(t))-\Gamma_{2}({ℰ}_{1}(t))|\;dt\;\;+\int_{0}^{x}|\beta (x,t)|\cdot |\Gamma_{1}({ℰ}_{2}(t))-\Gamma_{2}({ℰ}_{2}(t))|\;dt.$$

Since ${ℰ}_{1}$ and ${ℰ}_{2}$ are Banach contraction mappings with constants $\kappa_{1}$ and $\kappa_{2}$,


$$\left|\Gamma_{1}({ℰ}_{1}(t))-\Gamma_{2}({ℰ}_{1}(t))|\leq \kappa_{1}\|\Gamma_{1}-\Gamma_{2}\right\|$$

and


$$|\Gamma_{1}({ℰ}_{2}(t))-\Gamma_{2}({ℰ}_{2}(t))|\leq \kappa_{2}\|\Gamma_{1}-\Gamma_{2}\|.$$

Therefore,


$$|(T\Gamma_{1})(x)-(T\Gamma_{2})(x)|\leq \int_{0}^{x}\kappa_{1}\|\Gamma_{1}-\Gamma_{2}\|+\kappa_{2}\|\Gamma_{1}-\Gamma_{2}\|dt.$$

This simplifies to


$$|(T\Gamma_{1})(x)-(T\Gamma_{2})(x)|\leq \left(\kappa_{1}+\kappa_{2}\right)\|\Gamma_{1}-\Gamma_{2}\|x.$$

Taking the supremum over x∈[0,1],


$$\|T\Gamma_{1}-T\Gamma_{2}\|\leq (\kappa_{1}+\kappa_{2})\|\Gamma_{1}-\Gamma_{2}\|=\varphi (\|\Gamma_{1}-\Gamma_{2}\|).$$

Thus, the operator *T* satisfies the contraction condition with respect to the function φ.

Since $\kappa_{1}+\kappa_{2}<1$, we have $\varphi (t)=(\kappa_{1}+\kappa_{2})t<t$ for all *t* > 0. This shows that the mapping φ satisfies the condition for the Matkowski Fixed Point Theorem (1).

By the Matkowski fixed point theorem, there exists a unique mapping Γ∈𝒳 such that TΓ=Γ. This Γ is the unique solution to the functional equation Eq (8).

**Note.** The choice of Matkowski’s fixed point theorem is essential because the operator in (8), due to its integral form and the dependence of α(x,t) and β(x,t) on both *x* and *t*, does not generally satisfy the uniform contraction requirement *k* < 1 required by the Banach contraction principle. Matkowski’s theorem allows weaker contractive conditions through a comparison function φ with $\varphi^{\left(n\right)}(t)\to 0$ as n→∞, enabling us to establish existence and uniqueness where classical contraction methods fail.

**Corollary 1.** *Consider the functional equation* Eq (8)*. Suppose that ${ℰ}_{1},{ℰ}_{2}$ are Banach Contraction mappings with contraction coefficients $\kappa_{1},\kappa_{2}$ with $\kappa_{1}\leq \kappa_{2}$ and satisfy $2\kappa_{2}<1.$ Then* Eq (8) *has a unique solution. Moreover, the sequence $\left\{\Gamma_{n}\right\}$ in 𝒳 defined for each x∈[0,1] by*


$$\Gamma_{n}(x)=\int_{0}^{x}\alpha (x,t)\Gamma_{n}({ℰ}_{1}(t))+\beta (x,t)\Gamma_{n}({ℰ}_{2}(t))dt,$$

*for all n∈ℕ, where $\Gamma_{0}$ is given in 𝒳, converges to a unique solution of* Eq 8.

The previously mentioned mathematical model is utilized to evaluate the training of rewards, addressing the limited challenges of reinforcement, routine instruction, rapid improvement within operant-control theory. In the next section, we will focus on specific experiments.

## Background and motivation

The following two behavioral experiments strongly motivate our modelling framework by demonstrating that learning in operant-control systems is an adaptive, iterative process where response probabilities evolve continuously with experience. Skinner’s pigeon experiment and the human fMRI reward study both show that behavior is dynamically reshaped by reward history and stimulus predictability, revealing probabilistic adjustments far more complex than those captured by traditional discrete reinforcement models. These empirical insights justify the need for our integral functional equation approach, which models probability evolution as a continuous process and supports rigorous analysis via Matkowski’s fixed point theorem.

### Experiment I: Pigeon experiment

In 1943, behavioral psychologist Skinner embarked on a seemingly far-fetched idea: using trained pigeons to guide armed missiles toward enemy targets [26]. At that time, radar had not been invented, and the mechanical equipment needed to guide a missile left little room for explosives.

Skinner [27] developed a technique known as shaping. He would provide food rewards to his subjects when they approximated the desired behavior. Gradually, as they moved closer to the goal, Skinner adjusted the rewards to be more specific, eventually guiding the subjects to perform exactly as he intended.

**Fig 1:**
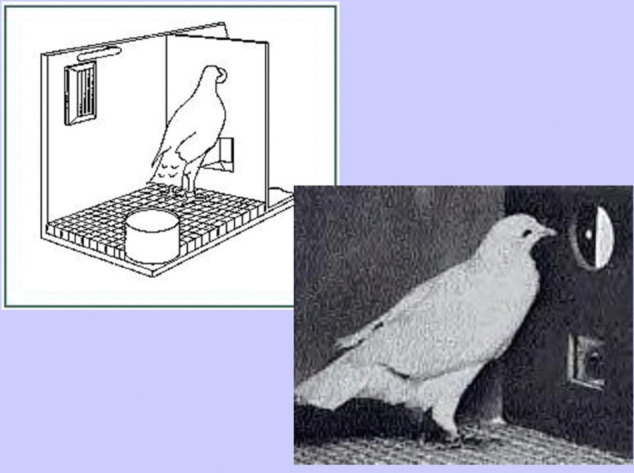
Pigeon project chamber.

He began this project by showing pigeons a dot projected on a translucent screen. Initially, a pigeon approaching the screen was rewarded with food. Over time, Skinner rewarded the bird for pecking closer and closer to the dot until it was precisely pecking on the target. Eventually, he moved the dot back and forth, and the pigeon learned to follow it quickly and accurately, ultimately training to track a moving battleship. Here below, Fig 1 illustrates the experimental setup.

It performed exceptionally well in tests for the Navy. The pigeons accurately guided the missile, demonstrating the effectiveness of this innovative guidance system.

In this experiment, the event that changes the probability is assumed to correspond perfectly with the pigeon’s response. We assume that, at any given time during the experiment, the probabilities of the pigeon pecking and not pecking the dot are *x* and 1–*x*, respectively. It is further assumed that, in the next trial, either response will alter the probability of pecking. This modification is modeled by continuous mappings on [0,1]. When the pigeon pecks the dot, the probability in the next trial changes at the rate ${ℰ}_{1}(x)$, whereas if it does not peck the dot, the probability changes at the rate ${ℰ}_{2}(x)$.

It can be described by the following functional equation:

$$\Gamma (x)=\int_{0}^{x}t\Gamma ({ℰ}_{1}(t))+(1-t)\Gamma ({ℰ}_{2}(t))dt$$ (10)

for all x∈[0,1], where ${ℰ}_{1}$ and ${ℰ}_{2}$ are continuous mappings from [0,1] to [0,1], and Γ:[0,1]→ℝ is the unknown mapping with the initial condition Γ(0)=0.

In this model, Γ represents the probability iteration mapping, which describes how the probability of the pigeon pecking evolves over successive trials. The condition Γ(0)=0 reflects the assumption that at the beginning of the experiment (before any trial is conducted), the initial probability of pecking is zero.

### Experiment II : The predictability of rewards influences human behavior

An adult participant underwent fMRI scanning while receiving small amounts of either fruit juice or water orally [28]. During the scan, the subject received these liquids in either a predictable or unpredictable manner across two separate sessions.

**Fig 2:**
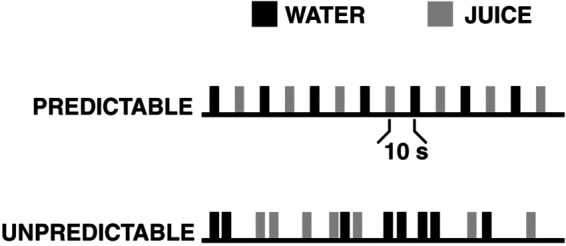
Design of fMRI experiment.

In the predictable session, fruit juice and water were alternated every 10 seconds at a fixed interval. In the unpredictable session, the order of fruit juice and water was randomized, and the interval between stimuli was also randomized based on a Poisson distribution with an average of 10 seconds [29]. Here below, Fig 2 illustrates the design of an experiment.

The subject received 0.8 ml of both fruit juice and water through two plastic tubes. A mouthpiece held the tubes in place over the tongue, with fruit juice delivered on the left side and water on the right side.

Four conditions representing the four possible events are given by the following Table 1. Here, by the work discussed in [7], we propose the following general equation:

$$\Gamma (x)=\int_{0}^{x}pt\Gamma ({ℰ}_{1}(t))+(1-p)t\Gamma ({ℰ}_{2}(t))+p(1-t)\Gamma ({ℰ}_{3}(t))\;+(1-p)(1-t)\Gamma ({ℰ}_{4}(t))dt$$ (11)

**Table 1:** Corresponding probabilities and operators.

Outcomes	Probability	Operator
predictable–preferred fluid	*px*	$\varepsilon_{1}(x)$
predictable–non preferred fluid	(1–*p*)*x*	$\varepsilon_{2}(x)$
unpredictable–preferred fluid	*p*(1–*x*)	$\varepsilon_{3}(x)$
unpredictable–non preferred fluid	(1–*p*)(1–*x*)	$\varepsilon_{4}(x)$

for all x∈[0,1], p∈[0,1], where ${ℰ}_{1}$, ${ℰ}_{2}$, ${ℰ}_{3}$ and ${ℰ}_{4}$ are continuous mappings from [0,1] to [0,1], and Γ:[0,1]→ℝ is the unknown mapping with the initial condition Γ(0)=0.

In this model, Γ denotes the probability of receiving the preferred liquid over successive trials. The initial condition Γ(0)=0 reflects the assumption that, at the start of the experiment (before any trial is conducted), the probability of obtaining the preferred liquid is zero.

**Remark 1.** Note that the boundary value Γ(0)=0 holds automatically for the integral equation. Thus, unlike the discrete form in [25] (where conditions such as ${ℰ}_{3}(0)={ℰ}_{4}(0)=0$ are often imposed to guarantee Γ(0)=0), no extra initial conditions on the values ${ℰ}_{i}(0)$ are needed here.

**Remark 2.** In the particular form (10), the weights of the two responses (*t* and 1–*t*) depend only on *t*, meaning the contribution of $\Gamma ({ℰ}_{1}(t))$ and $\Gamma ({ℰ}_{2}(t))$ to the integral changes linearly with *t*.

In the generalized form Eq (8), α(x,t) and β(x,t) are allowed to depend on both *x* and *t*, not just *t*. This means the weighting between the two responses can vary with respect to the current state *x* as well as the trial variable *t*.

Thus, Eq (8) generalizes Eq (11) by introducing more flexible and realistic weighting mappings that can capture more complex behavioral or probabilistic dependencies between *x* and *t*.

## Illustrative examples

Next, we present two illustrative examples that demonstrate the validity of the hypotheses and the practical utility of our results.

**Example 1.** *Consider the functional equation*

$$\Gamma (x)=\int_{0}^{x}t\Gamma ({ℰ}_{1}(t))+(1-t)\Gamma ({ℰ}_{2}(t))dt.$$ (12)

*for all x∈[0,1] and define the mappings ${ℰ}_{1}$ and ${ℰ}_{2}$ as follows:*


$${ℰ}_{1}(x)=\frac{x+1}{4},\;{ℰ}_{2}(x)=\frac{x+2}{5}.$$

These mappings satisfy ${ℰ}_{1}(0)=\frac{1}{4}$ and ${ℰ}_{2}(0)=\frac{2}{5}$, so ${ℰ}_{1}(0)\neq 0$ and ${ℰ}_{2}(0)\neq 0$.

Also, both ${ℰ}_{1}$ and ${ℰ}_{2}$ are Banach contractions mappings, Since ${ℰ}_{1}(x)=\frac{x+1}{4}$, we have


$$|{ℰ}_{1}(x)-{ℰ}_{1}(y)|=\frac{\left|x-y\right|}{4}.$$

So the contraction coefficient is $\kappa_{1}=\frac{1}{4}$.

For ${ℰ}_{2}(x)=\frac{x+2}{5}$, we have


$$|{ℰ}_{2}(x)-{ℰ}_{2}(y)|=\frac{\left|x-y\right|}{5}.$$

So the contraction coefficient is $\kappa_{2}=\frac{1}{5}$.

The sum of the contraction coefficients is:


$$\kappa_{1}+\kappa_{2}=\frac{1}{4}+\frac{1}{5}=\frac{5}{20}+\frac{4}{20}=\frac{9}{20}<1.$$

Thus, the condition $\kappa_{1}+\kappa_{2}<1$ is satisfied. Since all the assumptions of Theorem 2 hold, the functional equation Eq (12) has a unique solution.

We define the sequence $\Gamma_{n}(x)$ starting with $\Gamma_{0}(x)=x$ and compute each successive iteration using the recursive formula


$$\Gamma_{n+1}(x)=\int_{0}^{x}t\Gamma_{n}\left(\frac{t+1}{4}\right)+(1-t)\Gamma_{n}\left(\frac{t+2}{5}\right)\;dt.$$

Since $\Gamma_{0}(x)=x$, for the first iteration $\Gamma_{1}(x)$, we have


$$\Gamma_{1}(x)=\int_{0}^{x}\left(t\cdot \frac{t+1}{4}+(1-t)\cdot \frac{t+2}{5}\right)\;dt.$$

This simplifies to


$$\Gamma_{1}(x)=\int_{0}^{x}\left(\frac{t(t+1)}{4}+\frac{\left(1-t)(t+2\right)}{5}\right)\;dt.$$

Breaking this down further, we compute the terms


$$\frac{t(t+1)}{4}=\frac{t^{2}+t}{4},\;\frac{\left(1-t)(t+2\right)}{5}=\frac{t+2-t(t+2)}{5}.$$

Therefore


$$\Gamma_{1}(x)=\int_{0}^{x}\left(\frac{t^{2}+t}{4}+\frac{t+2-t(t+2)}{5}\right)\;dt.$$

Simplifying further and computing this integral gives the first iteration result.


$$\Gamma_{1}(x)=\frac{x^{3}}{60}+\frac{x^{2}}{40}+\frac{2x}{5}.$$

Next, for the second iteration $\Gamma_{2}(x)$, we use the recursive formula


$$\Gamma_{2}(x)=\int_{0}^{x}t\Gamma_{1}\left(\frac{t+1}{4}\right)+(1-t)\Gamma_{1}\left(\frac{t+2}{5}\right)\;dt.$$

Substitute the expression for $\Gamma_{1}(x)$ into this equation and compute the integral.


$$\Gamma_{2}(x)=\frac{61x^{5}}{2400000}+\frac{13x^{4}}{76800}+\frac{3217x^{3}}{480000}+\frac{3577x^{2}}{320000}+\frac{619x}{3750}.$$

Finally, for the third iteration $\Gamma_{3}(x)$, we have


$$\Gamma_{3}(x)=\int_{0}^{x}t\Gamma_{2}\left(\frac{t+1}{4}\right)+(1-t)\Gamma_{2}\left(\frac{t+2}{5}\right)\;dt.$$

$$\Gamma_{3}(x)=\frac{128161x^{7}}{53760000000000}+\frac{1694537x^{6}}{23040000000000}+\frac{39825883x^{5}}{3840000000000}+....$$

**Fig 3:**
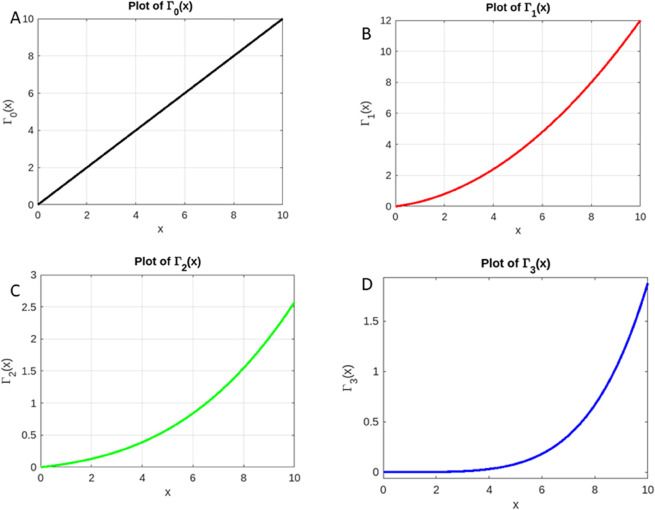
Sequential evolution of the iterative process. Illustration of the Picard iterative scheme applied to the operator *T*. (A): Initial approximation $\Gamma_{0}$. (B): Picard iterate $\Gamma_{1}=T\Gamma_{0}$. (C): Picard iterate $\Gamma_{2}=T\Gamma_{1}$. (D): Picard iterate $\Gamma_{3}=T\Gamma_{2}$.

After three iterations, we observe that the sequence $\left\{\Gamma_{n}\right\}$ is converging towards a fixed point mapping. The sequence continues to get closer to the unique solution of the functional equation as *n* increases, demonstrating the convergence guaranteed by Theorem 2. In the following, Fig 3 illustrates the first three iterations.

**Example 2.** *Consider the functional equation defined as follows:*

$$\Gamma (x)=\int_{0}^{x}pt\Gamma ({ℰ}_{1}(t))+(1-p)t\Gamma ({ℰ}_{2}(t))+p(1-t)\Gamma ({ℰ}_{3}(t))\;+(1-p)(1-t)\Gamma ({ℰ}_{4}(t))dt$$ (13)

*Let ${ℰ}_{1},{ℰ}_{2},{ℰ}_{3},{ℰ}_{4}$ be defined as follows:*


$${ℰ}_{i}(t)=\frac{t}{5}+\frac{1}{5}\;\text{for }i=1,2,3,4.$$

Each of these mappings does not vanish at *t* = 0


$${ℰ}_{i}(0)=\frac{1}{5}\text{ for }i=1,2,3,4.$$

To determine the contraction coefficients, we compute


$$|{ℰ}_{i}(x)-{ℰ}_{i}(y)|=\frac{\left|x-y\right|}{5}.$$

Thus, the contraction coefficient for each mapping ${ℰ}_{i}$,


$$\kappa_{i}=\frac{1}{5}\text{ for }i=1,2,3,4.$$

Summing the contraction coefficients yields


$$\kappa_{1}+\kappa_{2}+\kappa_{3}+\kappa_{4}=\frac{1}{5}+\frac{1}{5}+\frac{1}{5}+\frac{1}{5}=\frac{4}{5}.$$

Since $\frac{4}{5}<1$ and the total sum of the contraction coefficients is less than 1, the functional equation Eq (13) has a unique solution.

Let the initial mapping $\Gamma_{0}$ be given by


$$\Gamma_{0}(x)=x.$$

Given the iterative formula


$$\Gamma_{n}(x)=\int_{0}^{x}[pt\Gamma_{n-1}(\varepsilon_{1}(t))+(1-p)t\Gamma_{n-1}(\varepsilon_{2}(t))+p(1-t)\Gamma_{n-1}(\varepsilon_{3}(t))\;+(1-p)(1-t)\Gamma_{n-1}(\varepsilon_{4}(t))]\;dt.$$

Now, substitute ${ℰ}_{i}(t)$


$${ℰ}_{i}(t)=\frac{t}{5}+\frac{1}{5},$$

And the iteration formula becomes


$$\Gamma_{n}(x)=\int_{0}^{x}[pt\Gamma_{n-1}\;\left(\frac{t}{5}+\frac{1}{5}\right)+(1-p)t\Gamma_{n-1}\;\left(\frac{t}{5}+\frac{1}{5}\right)\;\;+\;p(1-t)\Gamma_{n-1}\;\left(\frac{t}{5}+\frac{1}{5}\right)+(1-p)(1-t)\Gamma_{n-1}\;\left(\frac{t}{5}+\frac{1}{5}\right)]\;dt.$$

Since $\Gamma_{n-1}\left(\frac{t}{5}+\frac{1}{5}\right)$ is the same for all terms,


$$\Gamma_{n}(x)=\int_{0}^{x}\left[(pt+(1-p)t+p(1-t)+(1-p)(1-t))\Gamma_{n-1}\left(\frac{t}{5}+\frac{1}{5}\right)\right]\;dt.$$

$$\Gamma_{n}(x)=\int_{0}^{x}\left[t+(1-t)\right]\Gamma_{n-1}\left(\frac{t}{5}+\frac{1}{5}\right)\;dt.$$

$$\Gamma_{n}(x)=\int_{0}^{x}\Gamma_{n-1}\left(\frac{t}{5}+\frac{1}{5}\right)\;dt.$$

Starting with $\Gamma_{0}(x)=x$


$$\Gamma_{1}(x)=\int_{0}^{x}\left(\frac{t}{5}+\frac{1}{5}\right)\;dt.$$

Calculate the integral


$$\Gamma_{1}(x)=\int_{0}^{x}\frac{t}{5}\;dt+\int_{0}^{x}\frac{1}{5}\;dt.$$

$$\Gamma_{1}(x)=\frac{x^{2}}{10}+\frac{x}{5}.$$

We compute $\Gamma_{2}(x)$ using the iterative formula


$$\Gamma_{2}(x)=\int_{0}^{x}\Gamma_{1}\left(\frac{t}{5}+\frac{1}{5}\right)dt=\int_{0}^{x}\left(\frac{{\left(\frac{t}{5}+\frac{1}{5}\right)}^{2}}{10}+\frac{\frac{t}{5}+\frac{1}{5}}{5}\right)dt.$$

Simplifying the terms


$$\Gamma_{2}(x)=\int_{0}^{x}\left(\frac{t^{2}}{250}+\frac{2t}{250}+\frac{1}{250}+\frac{t}{25}+\frac{1}{25}\right)dt.$$

$$\Gamma_{2}(x)=\frac{x^{3}}{750}+\frac{6x^{2}}{750}+\frac{11x}{250}.$$

Now, for $\Gamma_{3}(x)$, we use the iterative formula


$$\Gamma_{3}(x)=\int_{0}^{x}\Gamma_{2}\left(\frac{t}{5}+\frac{1}{5}\right)dt.$$

Substitute $\Gamma_{2}\left(\frac{t}{5}+\frac{1}{5}\right)$


$$\Gamma_{3}(x)=\int_{0}^{x}\left(\frac{{\left(\frac{t}{5}+\frac{1}{5}\right)}^{3}}{750}+\frac{6{\left(\frac{t}{5}+\frac{1}{5}\right)}^{2}}{750}+\frac{11\left(\frac{t}{5}+\frac{1}{5}\right)}{250}\right)dt.$$

$$\Gamma_{3}(x)=\int_{0}^{x}\left(\frac{\frac{t^{3}}{125}+\frac{3t^{2}}{125}+\frac{3t}{125}+\frac{1}{125}}{750}+\frac{6\left(\frac{t^{2}}{25}+\frac{2t}{25}+\frac{1}{25}\right)}{750}+\frac{11\left(\frac{t}{5}+\frac{1}{5}\right)}{250}\right)dt.$$

**Fig 4:**
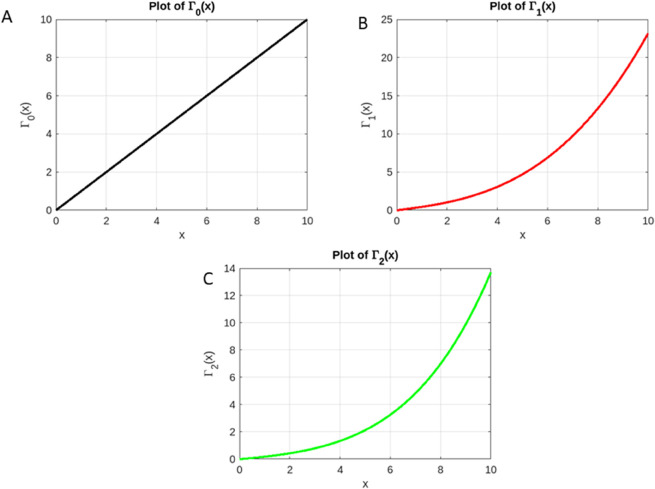
Sequential progression of Picard iterations demonstrating convergence toward the unique fixed point of *T*. (A): Initial approximation $\Gamma_{0}$. (B): Picard iterate $\Gamma_{1}=T\Gamma_{0}$. (C): Picard iterate $\Gamma_{2}=T\Gamma_{1}$.

We can continue expanding and integrating each term similarly to how it was done in the second iteration. In the following, Fig 4 illustrates the first two iterations.

## Conclusion

In this work, we presented a generalized functional equation Eq (8) inspired by operant-control experiments and established the existence and uniqueness of its solution using the Matkowski fixed point theorem under minimal assumptions. Our formulation extends earlier models based on behavioral probability learning and demonstrates that the integral form naturally encompasses previous discrete formulations as special cases. This theoretical advancement not only provides a stronger analytical foundation for studying adaptive and probabilistic behaviors but also broadens the applicability of functional equations in modeling complex dynamical systems. The approach highlights the versatility of fixed point theory as a unifying mathematical framework for problems encountered in behavioral sciences, control systems, and reinforcement-based learning models in engineering.

## Supporting information

S1 FileMinimal dataset for iterations $\Gamma_{0}$ to $\Gamma_{3}$.This CSV file contains the x-values in the first column and the corresponding values of $\Gamma_{0}(x)$, $\Gamma_{1}(x)$, $\Gamma_{2}(x)$, and $\Gamma_{3}(x)$ in subsequent columns. It allows replication of all iterative results and Figs 3 and 4 presented in the manuscript. Columns are labeled: x, Gamma0, Gamma1, Gamma2, Gamma3.(XLSX)
